# Factors associated with postpartum depression symptoms among postpartum women in five countries during the COVID-19 pandemic: an online cross-sectional study

**DOI:** 10.1186/s12888-023-04607-0

**Published:** 2023-03-15

**Authors:** Kelly Pereira Coca, Li-Yin Chien, Eun Young Lee, Ana Carolina de Prima Souza, Seo Ah Hong, Yan-Shing Chang

**Affiliations:** 1 Escola Paulista de Enfermagem, Universidade Federal de São Paulo, São Paulo, Brazil; 2 Institute of Community Health Care, College of Nursing, National Yang Ming Chiao Tung University, Yang-Ming Campus, Taipei, Taiwan; 3 Department of Nursing, Catholic Kkottongnae University, Cheongju, Republic of Korea; 4 ASEAN Institute for Health Development, Institute for Health and Society, Mahidol University, Hanyang University, Seoul, Republic of Korea; 5 Florence Nightingale Faculty of Nursing, Midwifery and Palliative Care, King’s College London, London, UK; 6 ASEAN Institute for Health Development, Mahidol University, Salaya, Thailand

**Keywords:** Postpartum depression, Food insecurity, Social support, Breastfeeding, SARS-CoV-2, Infant health, Mother health, Pregnancy

## Abstract

**Background:**

This study aimed to examine factors associated with postpartum depression (PPD) symptoms during the COVID-19 pandemic among postpartum women in five countries, a subject that has not been investigated thus far.

**Methods:**

A multi-country, cross-sectional, online survey was conducted with a convenience sample of 3,523 postpartum women in Brazil, South Korea, Taiwan, Thailand, and the United Kingdom, from July to November 2021. Sociodemographic and obstetric data, food insecurity, COVID-19 positive status, COVID-19 vaccination, infant feeding, breastfeeding belief score, and social support were investigated. PPD and social support were measured using the Edinburgh Postnatal Depression Scale and Maternal Social Support Scale, respectively. Descriptive statistics, chi-squared tests, and t-tests were used to identify associations with PPD symptoms. A binary logistic regression model was used to identify explanatory factors associated with PPD and adjusted odds ratios (OR) and 95% confidence intervals (CIs) were calculated.

**Results:**

Women in Taiwan (AOR = 0.5; 95%CI 0.34, 0.73) and Thailand (AOR = 0.68; 95%CI 0.46, 0.99) had a lower risk of PPD symptoms than those in Brazil. In addition, women with planned pregnancies had a lower risk of PPD (AOR = 0.74; 95%CI 0.60, 0.91). Younger women (AOR = 1.62; 95%CI 1.05, 2.51), health problems during pregnancy, delivery, or postpartum (AOR = 1.71; 95%CI 1.42, 2.06), and no change or worse food insecurity during COVID-19 (AOR = 1.66; 95%CI 1.21, 1.27 for no change and AOR = 1.68; 95%CI 1.27, 1.23, respectively) presented a higher likelihood of having PPD. Feeding babies with expressed human milk (AOR = 1.25; 95%CI 1.03, 1.50) and/or complementary food (AOR = 1.51; 95%CI 1.17, 1.94) were associated with PPD symptoms. Women who received low (AOR = 7.74; 95%CI 5.43, 11.03) or medium support (AOR = 3.25; 95%CI 2.71, 3.88) had higher likelihoods of PPD.

**Conclusion:**

PPD symptoms during the pandemic were high in young women, particularly Brazilian women, with health problems in the puerperal pregnancy cycle who fed their babies expressed breast milk and/or complementary food. Low social support also impacted PPD symptoms. This study highlights the need for the professional screening for PPD and provision of virtual or personal support.

## Background

The COVID-19 pandemic has affected mental health globally [1]. A survey conducted in the United Kingdom (UK) showed the effect of social isolation or social distancing on well-being. An increase in depression, anxiety, stress, and other negative feelings were identified, which may be related to individual, social, and population factors [2].

Specific populations, such as pregnant and breastfeeding women, have also been affected by the COVID-19 pandemic. A study conducted in Ireland, Norway, Switzerland, the Netherlands, and the UK showed that the prevalence of major depression symptoms (Edinburgh Depression Scale ≥ 13) was 15% in pregnant women and 13% in breastfeeding women up to three months postpartum. In addition, the authors identified moderate and severe generalized anxiety symptoms in 11% and 10% of the patients, respectively [3].

PPD is the most common psychological condition following delivery [4]. It can start at any time after childbirth within the first year, and continue for many years [4, 5]. The global prevalence of PPD is 17.2% with South America being 21.7%, Northern Europe 13.8%, Eastern Asia 17.4%, Southern Asia 19.8%, and South-Eastern Asia 13.5% [6].

The prevalence of PPD is very high in some countries and might be due to cross-cultural variables, biological vulnerability factors, and different socio-economic environments, such as levels of social support and stress [7]. Nations with a higher prevalence of PPD have a higher rates of income inequality, higher maternal mortality, infant mortality, or women of childbearing age working 40 h or more/week [8]. High levels of depressive symptoms and anxiety in pregnant and breastfeeding women during the COVID-19 pandemic were found to be associated with chronic mental illness, chronic postpartum somatic illness, and unplanned pregnancy [3]. Low social support was also a predictor of PPD [9].

Women with PPD are less sensitive to their infants and more negative about their infant experience. They can also present with disturbances in early mother-infant interactions and are associated with poorer infant cognitive outcomes at 18 months [10]. It may also have a negative impact on breastfeeding initiation and duration [11, 12]. Women in conflict with partners are associated with a higher risk of PPD [13]. Early life abuse, adult abuse, maternal low education, low socioeconomic status at the time of pregnancy, and lack of social support have been consistently identified as risk factors for PPD in low- and middle-income countries [14]. However, there is a lack of research on the association between breastfeeding and PPD, and the association between COVID-19 related factors and PPD.

The lockdown periods and limited social contact and social support during the COVID-19 pandemic have been a major challenge for mothers, children, and families [15]. Investigating the factors causing PPD symptoms during the pandemic could help healthcare professionals and policymakers understand the situation and provide strategies to support postpartum women with PPD.

This study aimed to examine the factors associated with postpartum depression symptoms during the COVID-19 pandemic among postpartum women in five countries.

## Methods

### Study design and Sites

Data from a multi-country, cross-sectional, online survey on postpartum women’s COVID-19-breastfeeding practices, including vaccination and postpartum depression, were collected in five countries: Brazil, South Korea, Taiwan, Thailand, and the UK. The analysis focused on postpartum depression and associated factors in postpartum women during the COVID-19 pandemic between July 2021 and November 2021.

### Population and data collection

We considered postpartum women who were 18 to 49 years old (Taiwan: between 20 and 49 years old), literate in their country’s official language, and up to six months postpartum as criteria of inclusion. Women who did not live in one of the study countries during the survey period were excluded. To ensure participants were postpartum women, before filling in the questionnaire, they had to confirm: “I gave birth in the past 6 months” and “I understand that I must not take part if I do not meet the criteria for participation”.

A questionnaire in the local languages of participating countries (Portuguese, Korean, Chinese, Thai, and English) was used to collect information after being created in English, translated into the local language, and back-translated into English.

The Google Forms survey link was distributed via email posted on social media platforms (Instagram, Facebook, Twitter, and WhatsApp) and sent by personal networks, health professional groups, and nonprofit organizations.

Data collection proceeded after obtaining ethics approval from five respective in-country universities’ Ethics Committees. The participants were invited to complete the questionnaire after voluntarily signing an online informed consent form.

### Measures of variables

Postpartum depression was measured by self-reported major depressive symptoms using the Edinburgh Postnatal Depression Scale (EPDS), which screens for symptoms of perinatal depression and anxiety. The self-report instrument assesses emotional experiences over the past seven days using a 10-item Likert scale. Each item uses a four-point scale (from zero to three) and has a total range of 0–30 [16]. A score of 13 or more was considered a postpartum depression “case.” We used validated EPDS versions from each country [17–20].

We also collected data on age, educational level, working status, marital status, local residence, parity, planned pregnancy, delivery mode, birth weight, health problems of mother during pregnancy/delivery or postpartum number of postnatal care visits, and food insecurity (if it changed from before and during the COVID-19 pandemic), if women were tested as COVID-19 positive, received COVID-19 vaccinations, and social support. To identify the variable “health problems of mother” women responded “Yes” or “No” for each question: “Do you have any health problems during pregnancy?”, “Do you have any health problems during delivery?” and “Do you have any health problems during postpartum?”.

Infant feeding information was collected using the question: “How was your youngest baby fed in the last 24 hours?” Women responded “Yes” or “No” for each item pertaining to the previous 24 h: breastfeeding (baby only fed directly from the breast), expressed human milk, infant formula feed, and solid/semi-solid or soft foods (including non-breast milk liquids). Further, beliefs towards breastfeeding scores (from 6 to 18) were obtained using a 3-point Likert scale (1 = agree, 2 = uncertain, 3 = disagree) in response to the following questionnaire statements:1) “COVID-19 can be passed on to the baby through human milk and breastfeeding;” 2) “If the mother is confirmed or suspected to have the COVID-19 infection, the mother should not breastfeed;” 3) “If the mother is confirmed or suspected to have the COVID-19 infection, the baby should still be immediately be placed skin-to-skin and breastfed following delivery;” 4) “If the mother is confirmed or suspected of having the COVID-19 infection, it is safer to give the baby infant formula milk compared to human milk or practice breastfeeding at the breast;” 5) “A breastfeeding mother who is confirmed or suspected of having the COVID-19 infection should always wear a face mask when breastfeeding;” and 6) “A mother who is confirmed or suspected to have the COVID-19 infection can touch and hold her newborn baby without wearing a face mask.” Questions 3 and 5 were coded before being summed up in reverse. A higher score indicated a more positive breastfeeding belief [21].

Social support was measured using the Maternal Social Support Scale (MSSS). It is a six-item self-report measure of maternal perceptions of social support: (1) “I have good friends who support me,” (2) “My family is always there for me,” (3) “My husband/partner helps me a lot,” (4) “There is conflict with husband/partner,” (5) “I feel controlled by my husband/partner,” and (6) “I feel loved by my husband/partner.” Each item was rated on a 5-point Likert scale from 1 (never) to 5 (always), scoring up to 30 points. Items 4 and 5 were reverse-scored [22]. We considered low social support scores to be < 19, medium social support scores to be 19–24, and high social support scores to be 25–30 [22].

### Statistical analysis

Data were analyzed using SAS 9.3 (SAS Institute Inc., Cary, NC, USA). Descriptive statistics were used to calculate frequencies and percentages for categorical variables and means and standard deviations for continuous variables. The chi-square test or Student’s t-test was used to examine the association between the independent variables and PPD symptoms (13 or higher scores), as appropriate. Co-variates with p-value < 0.05 in univariate analysis were entered into final multiple logistic regression. A binary logistic regression model was used to identify explanatory factors associated with PPD symptoms, and crude and adjusted odds ratios (COR and AOR) and 95% confidence intervals (CIs) were calculated. Statistical significance was set at a p-value < 0.05. For multiple linear regression, independent variables which showed p-value less than 0.05 in simple linear regression were employed in multiple linear regression.

## Results

A total of 3,253 women participated in the survey (Brazil: 560; Taiwan: 614; Thailand: 840; South Korea: 381; UK: 858). Participants were mostly 30–39 years old (61.6%), only 24.2% had a college or secondary education level, more than half were on maternity leave (59.2%), mostly married (95.5%), and lived in an urban area residence (72.6%). Regarding obstetric data, 57% had one child, most had a planned pregnancy (80.4%), 39% had a C-section delivery mode, only 32.9% chose 4 or more postnatal care factors, only 12.3% had a preterm delivery, and 43.7% experienced maternal health problems (during pregnancy, delivery, or postpartum period) (Table 1).

Table 1 shows 29.3% of women with postpartum depression symptoms based on the cut-off score (13 or more) and was associated with women being younger (p < .0001), with college or lower educational levels (p = .001), unemployed (p < .0001), unplanned pregnancies (p < .0001), health problems during pregnancy, delivery, or postpartum (p < .0001), and not receiving postnatal care support (p < .0001). Women whose food insecurity did not change or worsen during the COVID-19 pandemic were associated with PPD symptoms (p < .0001), and women with PPD symptoms tended not to receive the vaccination (p = .0177).

**Table 1:** Factors associated PPD symptoms with social demographic and obstetric women’s data in Brazil, South Korea, Taiwan, Thailand, and the UK during the COVID-19 pandemic (N = 3253)

Variables	Total		PPD symptoms				
			Yes		No		
	N	(%)	n	(%)	n	(%)	*P* value
**Total**	3253	(100.0)	954	(29.3)	2299	(70.7)	
**Social demographic data**							
*Age (years)*							
18–29	1094	(33.6)	383	(40.2)	711	(30.9)	**< 0.0001**
30–39	2005	(61.6)	534	(56.0)	1471	(64.0)	
41–49	154	(4.8)	37	(3.8)	117	(5.1)	
*Educational level*							
Secondary or lower	787	(24.2)	292	(30.6)	495	(21.5)	**< 0.0001**
University or higher	2465	(75.8)	662	(69.4)	1803	(78.5)	
Missing	1	(0.0)	0	(0.0)	1	(0.0)	
*Working status*							
Working	564	(17.3)	185	(19.4)	379	(16.5)	**< 0.0001**
On maternity leave	1926	(59.2)	506	(53.0)	1420	(61.8)	
Homemaker/unemployed	762	(23.5)	263	(27.6)	499	(21.7)	
Missing	1	(0.0)	0	(0.0)	1	(0.0)	
*Marital status*							
Married	3105	(95.5)	879	(92.1)	2226	(96.8)	**< 0.0001**
Others	148	(4.5)	75	(7.9)	73	(3.2)	
*Residence local*							
Urban	2360	(72.6)	673	(70.6)	1687	(73.4)	0.0916
Rural	892	(27.4)	281	(29.4)	610	(26.6)	
Missing	2	(0.0)	0	(0.0)	2	(0.0)	
**Obstetric data**							
*Parity*							
Primiparous	1853	(57.0)	568	(59.6)	1285	(55.9)	0.0535
2 or more	1398	(43.0)	386	(40.4)	1012	(44.1)	
Missing	2		1		1		
Planned pregnancy	2614	(80.4)	697	(73.1)	1917	(83.4)	**< 0.0001**
C-Section delivery mode	1268	(39.0)	357	(37.4)	911	(39.6)	0.2405
Preterm delivery	399	(12.3)	120	(12.6)	279	(12.1)	0.7259
*Birthweight*							
Low (< 2.5 kg)	250	(7.7)	73	(7.7)	177	(7.7)	0.9609
2.5 or more	3002	(92.3)	881	(92.3)	2121	(92.3)	
Missing	1	(0.0)	0	(0.0)	1	(0.0)	
Had health problem during pregnancy, delivery, or postpartum	1422	(43.7)	469	(49.2)	953	(41.5)	**< 0.0001**
Chose 4 or more postnatal support factors	1275	(39.2)	316	(33.1)	959	(41.7)	**< 0.0001**
**Impact of COVID-19**							
*Changes in food insecurity*							
No change (insecure - insecure)	298	(9.2)	132	(13.9)	166	(7.2)	**< 0.0001**
Worse	340	(10.5)	144	(15.1)	196	(8.5)	
Better	27	(0.8)	9	(0.9)	18	(0.8)	
No change (secure - secure)	2584	(79.5)	668	(70.1)	1916	(83.5)	
Missing	4	(0.0)	1	(0.0)	3	(0.0)	
Ever tested as COVID-19 positive	417	(12.8)	123	(12.9)	294	(12.8)	0.9351
COVID-19 vaccination (yes)	2348	(72.2)	661	(69.3)	1687	(73.4)	**0.0177**

Missing values were excluded (not counted) in all analyses.

Table 2 shows that most infants were breastfed at the breast in the 24 h prior to the survey (73.5%), 38.3% received expressed human milk, and 40.6% received formula feeding. Most postpartum women received professional healthcare support (67.1%), 51.6% also received support from a spouse/partner, friend, or relative, and 6.24% expressed high social support.

Women who expressed human milk (p = .0288) and gave complementary food to infants (p < .0001) were associated with PPD symptoms. Women who presented with PPD symptoms had lower mean scores on beliefs about breastfeeding (7.10; vs. 7.48; p = .0003). In addition, no support received for infant feeding (p < .0001), less health professional care postpartum feeding support (p = .0004), and less spouse/partner/friend or relative support (p < .0001) were associated with PPD symptoms. Low and middle social support were associated with postpartum depression (p < .0001) (Table 2).

**Table 2:** Infant feeding behavior and social support received in Brazil, South Korea, Taiwan, Thailand, and the UK during the COVID-19 pandemic (N = 3253)

	Total		PPD symptoms				
Variables			Yes		No		*P* value
	N	%	N	%	N	%	
Infant feeding behaviors*							
Breastfeeding**	2392	(73.5)	688	(72.1)	1704	(74.1)	0.2387
Expressed human milk	1246	(38.3)	393	(41.2)	853	(37.1)	**0.0288**
Infant formula	1321	(40.6)	411	(43.1)	910	(39.6)	0.0643
Solid, semi-solid or soft foods***	388	(11.9)	154	(16.1)	234	(10.2)	**< 0.0001**
**Belief towards breastfeeding** (Mean, SD)	7.37	2.66	7.10	2.64	7.48	2.67	**0.0003**
**Support for postnatal infant feeding**							
No support for infant feeding	505	(15.5)	196	(20.6)	309	(13.4)	**< 0.0001**
Healthcare professional support	2182	(67.1)	597	(62.6)	1585	(68.9)	**0.0004**
Spouse/partner, friend, or relative support	1678	(51.6)	440	(46.1)	1238	(53.9)	**< 0.0001**
Online support group	998	(30.7)	287	(30.1)	711	(30.9)	0.6352
**Social Support (MSSS)** (Missing = 4)							
Low social support (< 19 scores)	189	(5.8)	128	(13.4)	61	(2.7)	**< 0.0001**
Medium social support (19–24 scores)	1034	(31.8)	453	(47.4)	581	(25.3)	
High social support (25–30 scores)	2026	(62.4)	373	(39.1)	1653	(72.0)	

Missing values were excluded (not counted) in all analyses.

Breastfeeding Belief (range 0 to 12 scores).

*Mark all that apply.

**Breastfeeding = baby only fed directly at breast.

***Including non-human milk liquids.

The results of the logistic regression analysis are presented in Table 3. Women living in Taiwan and Thailand have a lower likelihood of PPD symptoms than those in Brazil. In addition, women who planned their pregnancies had a lower likelihood of PPD symptoms. Younger women who experienced health issues during pregnancy, delivery, or postpartum and experienced no change or worse food insecurity had a higher likelihood of PPD in the five countries. Feeding babies with expressed human milk and complementary food was associated with PPD symptoms. Women who received low or moderate support had a higher likelihood of having PPD symptoms. In Table 4 multiple linear regression confirm the results presented above.

**Table 3:** Logistic regression analysis of the factors associated with postpartum depression (N = 3523)

Variables	PPD symptoms (13 or more)					
	COR	(95% CI)		AOR	(95% CI)	
Country						
Taiwan	**0.70**	**(0.54-**	**0.90)**	**0.53**	**(0.37-**	**0.76)**
Thailand	1.10	(0.87-	1.38)	0.74	(0.52-	1.06)
South Korea	**1.35**	**(1.02-**	**1.78)**	0.99	(0.66-	1.50)
UK	0.83	(0.66-	1.05)	0.98	(0.73-	1.31)
Brazil	ref			ref		
**Social demographic data**						
*Age (years)*						
18–29	**1.70**	**(1.15-**	**2.52)**	**1.72**	**(1.12-**	**2.64)**
30–39	1.15	(0.78-	1.68)	1.36	(0.90-	2.05)
41–49	ref			ref		
Education (secondary or lower)	**1.61**	**(1.36-**	**1.90)**	1.09	(0.86-	1.37)
*Working status*						
Working	0.93	(0.74-	1.17)	1.12	(0.86-	1.47)
On maternity leave	**0.68**	**(0.56-**	**0.81)**	1.01	(0.80-	1.29)
Homemaker/unemployed	ref			ref		
Marital status (married)	**0.39**	**(0.28-**	**0.54)**	0.77	(0.52-	1.13)
**Obstetric data**						
Planned pregnancy	**0.54**	**(0.45-**	**0.65)**	**0.73**	**(0.59-**	**0.90)**
Health problem during pregnancy, delivery or postpartum	**1.37**	**(1.17-**	**1.59)**	**1.74**	**(1.45-**	**2.09)**
Received 4 or more postnatal care	**0.69**	**(0.59-**	**0.81)**	0.96	(0.78-	1.20)
**Impact of COVID-19**						
*Changes in food insecurity*						
No change (insecure - insecure)	**2.28**	**(1.79-**	**2.91)**	**1.63**	**(1.19-**	**2.22)**
Worse	**2.11**	**(1.67-**	**2.66)**	**1.68**	**(1.27-**	**2.21)**
Better	1.43	(0.64-	3.21)	1.15	(0.48-	2.75)
No change (secure - secure)	ref			ref		
COVID-19 vaccination (yes)	**0.82**	**(0.69-**	**0.97)**	1.15	(0.93-	1.42)
**Infant feeding**						
Expressed human milk	**1.19**	**(1.02-**	**1.39)**	**1.24**	**(1.03-**	**1.50)**
Solid, semi-solid or soft food	**1.70**	**(1.37-**	**2.12)**	**1.56**	**(1.22-**	**2.00)**
**Belief towards breastfeeding** (per one score increase)	**0.95**	**(0.92-**	**0.98)**	0.97	(0.93-	1.01)
**Support for postnatal infant feeding**						
No support for infant feeding	**1.67**	**(1.37-**	**2.03)**	1.30	(0.96-	1.74)
Healthcare professional support	**0.75**	**(0.64-**	**0.88)**	1.01	(0.81-	1.26)
Spouse/partner, friend, or relative support	**0.73**	**(0.63-**	**0.85)**	1.03	(0.85-	1.25)
**Social Support**						
Low social support (< 19 scores)	**9.30**	**(6.72-**	**12.87)**	**7.48**	**(5.26-**	**10.63)**
Medium social support (19–24 scores)	**3.46**	**(2.93-**	**4.08)**	**3.17**	**(2.66-**	**3.79)**
High social support (25–30 scores)	ref			ref		

Missing values were excluded (not counted) in all analyses.

COR: crude odds ratio, AOR: adjusted odds ratio, CI: confidence interval.

Bold values indicate statistical significance (p < .05).

Hosmer and Lemeshow Goodness-of-Fit Test = 7.72 and p-value = 0.461.

**Table 4:** Multiple linear regression analysis of the EPDS scores (N = 3523)

Variables	EPDS scores (Simple)				EPDS scores (Multiple)		
	*B*	*SE*	p	*B*		*SE*	p
Country							
Taiwan	-1.330	0.319	**< 0.0001**	-2.203		0.384	**< 0.0001**
Thailand	0.183	0.298	0.538	-1.142		0.384	**0.003**
South Korea	0.053	0.362	0.884	-1.323		0.422	**0.002**
UK	-0.426	0.297	0.151	0.150		0.316	0.635
Brazil	ref			ref			
**Social demographic data**							
Age (*Years*)							
18–29	1.950	0.468	**< 0.0001**	1.676		0.442	**0.000**
30–39	0.603	0.455	0.186	1.056		0.415	**0.011**
41–49	ref			ref			
Educational level (Secondary or lower)	1.447	0.223	**< 0.0001**	-0.007		0.249	0.979
*Working status*							
Working	-0.356	0.303	0.240	0.039		0.287	0.892
On maternity leave	-1.150	0.234	**< 0.0001**	-0.248		0.253	0.328
Homemaker/unemployed	ref			ref			
Marital status (married)	-3.172	0.458	**< 0.0001**	-0.651		0.435	0.135
Residence local (urban)	-0.435	0.215	**0.043**	0.014		0.220	0.949
**Obstetric data**							
Primiparous	0.460	0.194	**0.018**	0.421		0.187	**0.024**
Planned pregnancy	-1.826	0.240	**< 0.0001**	-0.852		0.229	**0.000**
C-Section delivery mode	-0.041	0.197	0.835				
Had preterm delivery	0.464	0.293	0.113				
Low birthweight (< 2.5 kg)	0.629	0.361	0.081				
Health problem during pregnancy, delivery or postpartum	1.064	0.193	**< 0.0001**	1.393		0.192	**< 0.0001**
Received 4 or more postnatal care	-0.950	0.196	**< 0.0001**	-0.046		0.225	0.839
**Impact of COVID-19**							
*Changes in food insecurity*							
No change (insecure - insecure)	3.016	0.328	**< 0.0001**	1.895		0.345	**< 0.0001**
Worse	2.557	0.310	**< 0.0001**	1.607		0.309	**< 0.0001**
Better	1.198	1.039	0.249	0.682		0.955	0.475
No change (secure - secure)	ref			ref			
Ever tested as COVID-19 positive	0.145	0.288	0.614				
COVID-19 vaccination (yes)	-0.221	0.214	0.304				
**Infant feeding**							
Breastfeeding direct at breast	-0.393	0.218	0.071				
Expressed human milk	0.445	0.198	**0.024**	0.615		0.197	**0.002**
Infant formula	0.424	0.196	**0.030**	0.453		0.197	0.021
Solid, semi-solid or soft foods	1.421	0.296	**< 0.0001**	1.021		0.273	**0.000**
**Belief towards breastfeeding** (per one score increase)	-0.120	0.036	**0.001**	-0.074		0.043	0.084
**Support for postnatal infant feeding**							
No support for infant feeding	1.407	0.264	**< 0.0001**	0.483		0.322	0.134
Healthcare professional support	-0.790	0.204	**0.000**	-0.078		0.230	0.733
Spouse/partner, friend, or relative support	-0.831	0.192	**< 0.0001**	-0.016		0.203	0.938
Online support group	0.263	0.208	0.207				
**Social Support**							
Low social support (< 19 scores)	6.589	0.387	**< 0.0001**	5.857		0.395	**< 0.0001**
Medium social support (19–24 scores)	3.466	0.195	**< 0.0001**	3.192		0.195	**< 0.0001**
High social support (25–30 scores)	ref			ref			
Intercept				8.485		0.996	**< 0.0001**
Adjusted R2	0.206			0.201			
F-value (p-value)	25.5		**< 0.0001**	31.2			**< 0.0001**

Missing values were excluded (not counted) in all analyses.

## Discussion

Pooled samples from Brazil, the UK, Taiwan, Thailand, and South Korea presented 29.3% of women with postpartum depression symptoms. We found that women who were 18–29 years old; experienced health problems during pregnancy, delivery, or postpartum; had worse or no change in food insecurity; low or middle social support; who fed their babies expressed human milk and/or complementary food; and had low or medium social support were factors associated with PPD symptoms during the COVID-19 pandemic among postpartum women in the five countries. Women who had PPD symptoms had lower belief towards breastfeeding. Women who planned their pregnancies had a low risk of developing PPD symptoms during the study period.

Our research supports studies reporting the prevalence of mental health issues during the COVID-19 pandemic [6, 23, 24], despite the difference in cut-off value between studies. We used the EPDS cut-off value 13, which, according to a systematic review, is more specific and could be used for postpartum women with higher symptom levels [25]. Considering that, rates of prevalence of PPD could be higher than studies using a cut-off value lower than 13. In clinical practice, health professionals may use a lower cut-off value to PPD screening.

Primiparous, women younger than 35 years, employed full-time, and middle-income categories increased the risk of depressive and anxiety symptoms during the outbreak [23]. A study in Japan identified a correlation between PPD and primiparity, premature delivery, difficult labor, concern about baby care, and experience of life events [26]. Similar results were found in the five countries in which PPD symptoms were associated with women being younger and who had health problems during pregnancy, delivery, or postpartum. Younger age and first baby experience may bring more anxiety about delivery and motherhood. Supporting these women might be effective to prevent and identify PPD earlier [26]. Healthcare professionals should discuss women’s mental health when caring for the health problems during pregnancy, delivery or postpartum [27].

Regarding food insecurity, a study identified an associated 253% higher risk of depression [28], results congruent with our findings that women who experienced worse or no change in food insecurity were associated with a higher risk of PPD symptoms. The COVID-19 pandemic has increased insecurity in women in many aspects. A scoping review conducted between 2020 and 2021 indicated an increased prevalence of food insecurity due to negative changes in food accessibility and availability [29]. Food insecurity compromises health because of its association with poor diet quality, obesity, depression, and high mortality rates [30]. It increases the risk of eating disorders, depression, and anxiety 1.19 times [31]. Maternal depression increases the risk of delayed early childhood development when associated with household food insecurity [32]. Therefore, it is important to identify and support households experiencing food insecurity to prevent and address maternal mental health problems.

Our study identified a higher likelihood of PPD symptoms in women who fed their baby human milk and/or complementary food. Another study conducted in the United States found that women who fed their children infant formula had 92% greater odds of PPD symptoms compared to breast or bottle feed with expressed human milk [33]. In addition, a recent systematic review showed that women who did not exclusively breastfeed had an 89% higher PPD rate [34].

Despite the pandemic giving mothers the opportunity to be at home to breastfeed their babies, first-time mothers may be at higher risk of breastfeeding cessation because of a lack of support [35]. Postpartum women need support to grow their babies, and the COVID-19 pandemic has negatively influenced their support. Mothers reported increased stress and isolation and had an immense desire for social and professional support [35]. Poor social support could be a reason for the increase in PPD symptoms found in our study.

A study of postpartum women in Canada showed that the COVID-19 pandemic interfered with access to formal and informal breastfeeding social support, which facilitated feelings of less protection and connectedness [36]. Supporting mothers during breastfeeding is important to clarify their doubts and discuss problems. Breastfeeding professional support increases the degree of mother’s satisfaction and helps them network, protecting breastfeeding rates for more than six months [37].

Health professional workers should screen for mental health issues and support women and their babies to protect them from negative consequences, especially in a pandemic such as COVID-19.

The limitations of the study are as follows: women completed the EPDS by themselves on an online survey form, which may impact the PPD symptom score. Only women who could access the internet participated in the study and might not be representative of different social classes. Non-probability samples may not be representative of all countries and cannot be generalized to other settings. Due to the study design, causation cannot be established in the cross-sectional.

## Conclusion

Postpartum depression symptoms were high and were associated with being younger, having health problems during pregnancy, delivery, and/or postpartum, worse and/or no change in food insecurity, low or middle social support, less professional care postpartum feeding support, and feeding babies with expressed human milk and complementary food during the COVID-19 pandemic.

The study highlights that professionals should be trained to identify potential postpartum women with PPD and provide personal or virtual support to guarantee adequate support during the COVID-19 pandemic. Supporting postpartum women’s mental health may protect the practice of breastfeeding and the well-being of newborns.

## Data Availability

All data generated or analyzed for this study are presented in the manuscript.

## Abbreviations

PPD: Postpartum depression
BF: Breastfeeding
EBF: Exclusive breastfeeding
UK: United Kingdom
WHO: World Health Organization
